# Identifying regions of disease-related variants in admixed populations with the summation partition approach

**DOI:** 10.1186/s12919-016-0018-9

**Published:** 2016-10-18

**Authors:** Jonathan Auerbach, Michael Agne, Rachel Fan, Adeline Lo, Shaw-Hwa Lo, Tian Zheng, Pei Wang

**Affiliations:** 1 Department of Statistics, Columbia University, 1255 Amsterdam Avenue, New York, NY 10027 USA; 2 Department of Political Science, University of California at San Diego, 9500 Gilman Drive, La Jolla, CA 92093 USA; 3 Department of Genetics and Genomic Sciences, Icahn School of Medicine at Mount Sinai, 1428 Madison Avenue, New York, NY 10029 USA

## Abstract

We propose a new method for identifying disease-related regions of single nucleotide variants in recently admixed populations. We use principal component analysis to derive both global and local ancestry information. We then use the summation partition approach to search for disease-related regions based on both rare variants and the local ancestral information of each region. We demonstrate this method using individuals with high systolic blood pressure from a sample of unrelated Mexican American subjects provided in the 19th Genetic Analysis Workshop.

## Background

Genome-wide association studies commonly use admixture mapping to search for disease-related regions in the genome of recently admixed populations. Admixture mapping refers to methods that trace the ancestral origin of genetic loci and then determine whether the ancestry providing that loci is in disequilibrium with the disease. These methods assume disease-related regions of the genome occur at different rates depending on whether they were inherited from one of the ancestral populations.

Hypertension is a serious disease, affecting 1 in 3 adult Americans and leading to over sixty thousand deaths in 2009 [1]. Yet, hypertension does not afflict Americans equally across race and ethnic lines. African Americans are twice as likely to have high blood pressure or to be taking antihypertensive medication than are Mexican Americans (~40 % vs. ~20 %) according to the Center for Disease Control [2]. The non-Hispanic white population is in the middle, with roughly 30 % having hypertension or being on antihypertensive medication.

Research also suggests individuals have a genetic predisposition to hypertension [3], and its disparate incidence across race and ethnic lines appears to make it a strong candidate for admixture mapping. While several methods exist for deriving this ancestral information, principal component analysis (PCA) has proven to be a simple, yet powerful, tool for reducing the variation of high-dimensional data and extracting ancestry related information from admixed samples [4, 5]. We use the loading scores from principal components to derive both global and local ancestral information for this reason.

Apart from admixture mapping, new research finds that rare variants have an important role in explaining complex diseases such as high blood pressure. We presume these rare variants contain additional disease-related information not present in common variants of the same region. The summation partition approach (SPA) [6] has been used successfully to collapse small groups of rare variants and investigate each group’s association with a disease. The advantage of SPA is that it can be more powerful than other collapsing techniques, especially when working with rare variants in relatively small data sets [6].

In this paper, we propose a new method, local ancestry–summation partition approach (LA-SPA), which combines the power of admixture mapping in leveraging local ancestry structures with the power of SPA in leveraging the information in rare variants. We demonstrate the LA-SPA method using individuals with high systolic blood pressure from the 19th Genetic Analysis Workshop (GAW19) Mexican American unrelated sample data set.

## Methods

We explore our LA-SPA method using GAW19 genome-wide association data from 1851 Mexican Americans [7]. Our data set contains 428,574 single-nucleotide variants (SNVs) from odd-numbered chromosomes. Missing data was relatively uncommon and therefore imputed by sampling uniformly over the remaining non-missing observations. Phenotype data on systolic blood pressure, diastolic blood pressure, year of examination, age, gender, and medication usage was also available. Medication status took 3 values depending on whether the individual was on hypertension medication, not on hypertension medication, or whether such a status was unknown. Systolic blood pressure is the dependent variable of this analysis.

Our method has 4 stages. In this section, we walk through each stage as we performed it on the GAW19 data.

### Stage 1: adjust for covariates and global population structure

We first obtain the residuals from regressing systolic blood pressure on age, gender, medication status, and the loading score of the first principal component of all 428,574 SNVs. These variables explain roughly 25 % of the variation of systolic blood pressure. The loading score represents global ancestry, and we only use the first principal component in this stage as additional components were deemed unnecessary (see Discussion below for more details). We interpret the residuals as containing information on systolic blood pressure in excess of confounding global variables. We treat the residuals as our new quantitative phenotype and denote it as *Y*.

### Step 2: divide variants into regions

We group consecutive SNVs into regions of 500. There were 862 regions in total. We then stratified the SNVs in each region into either “common” or “rare” SNV groups based on their minor allele frequency (MAF >0.05 or MAF <0.05). Roughly 80 % of SNVs in each region are rare variants.

### Step 3: calculate the local ancestry–summation partition approach statistic

We perform PCA on the group of common variants in each segment separately and recorded the values of the loading scores of the first 3 principal components. Local ancestry was estimated by performing the k-means algorithm (k = 3) on these components. We interpret the result as corresponding to the 3 possible ancestral origins of each region: white, African, and Native American. We then test the association between the adjusted phenotype from stage 1 and the rare SNV group of each segment by the local ancestral origin of the region using a variation of SPA. The following is a brief explanation of the SPA algorithm [6] and our variation.

Consider a region with *K* rare variants. The marginal SPA test statistic is defined as:

$$ {I}_1={\displaystyle \sum_{i=1}^K{n}_i^2}{\displaystyle {\left(\overline{Y_i}-\overline{Y}\right)}^2}, $$

where *n*_*i*_ is the total number of *i* ^*th*^ rare variants in all subjects, $\overline{Y_i}$ is the averaged phenotype for subjects having at least 1 rare variant at the *i* ^*th*^ SNV position and $\overline{Y}$ is the sample average. The value of *I*_1_ reflects the strength of the association between the group of rare variants and the residuals from stage 1.

To jointly model local ancestral information and rare variants, we propose an LA-SPA test statistic, *I*_*A*_, defined as:

$$ {I}_A={\displaystyle \sum_{j=1}^J{\displaystyle \sum_{i=1}^K{n}_{i,j}^2{\displaystyle {\left(\overline{Y_{i,j}}-\overline{Y}\right)}^2}}}, $$

where *n*_*i,j*_ is the total number of rare variants observed in subjects from *j* ^*th*^ local ancestry and $\overline{Y_{i,j}}$ is the mean phenotype of individuals with rare variants *i* in the *j* ^*th*^ local ancestral cluster, and $\overline{Y}$ is defined as before. *I*_*A*_ is a modification of *I*_1_, similar to the *I*_2_ of Fan [6]. It reflects the strength of the association between phenotypes and a region of rare variants, partitioned by the local ancestry of the region.

### Step 4: calculate *p* values by permutation

Permutation is used to evaluate the significance of each test statistic. For *I*_*A*_, 2 *p* values are generated reflecting permutation within and between ancestral origins. We refer to them as the global *p* value, which reflects information between common and rare variants, and the local *p* value, which reflects information within the rare variants grouped by the common variants. We used 10,000 permutations.

## Results

**Fig. 1:**
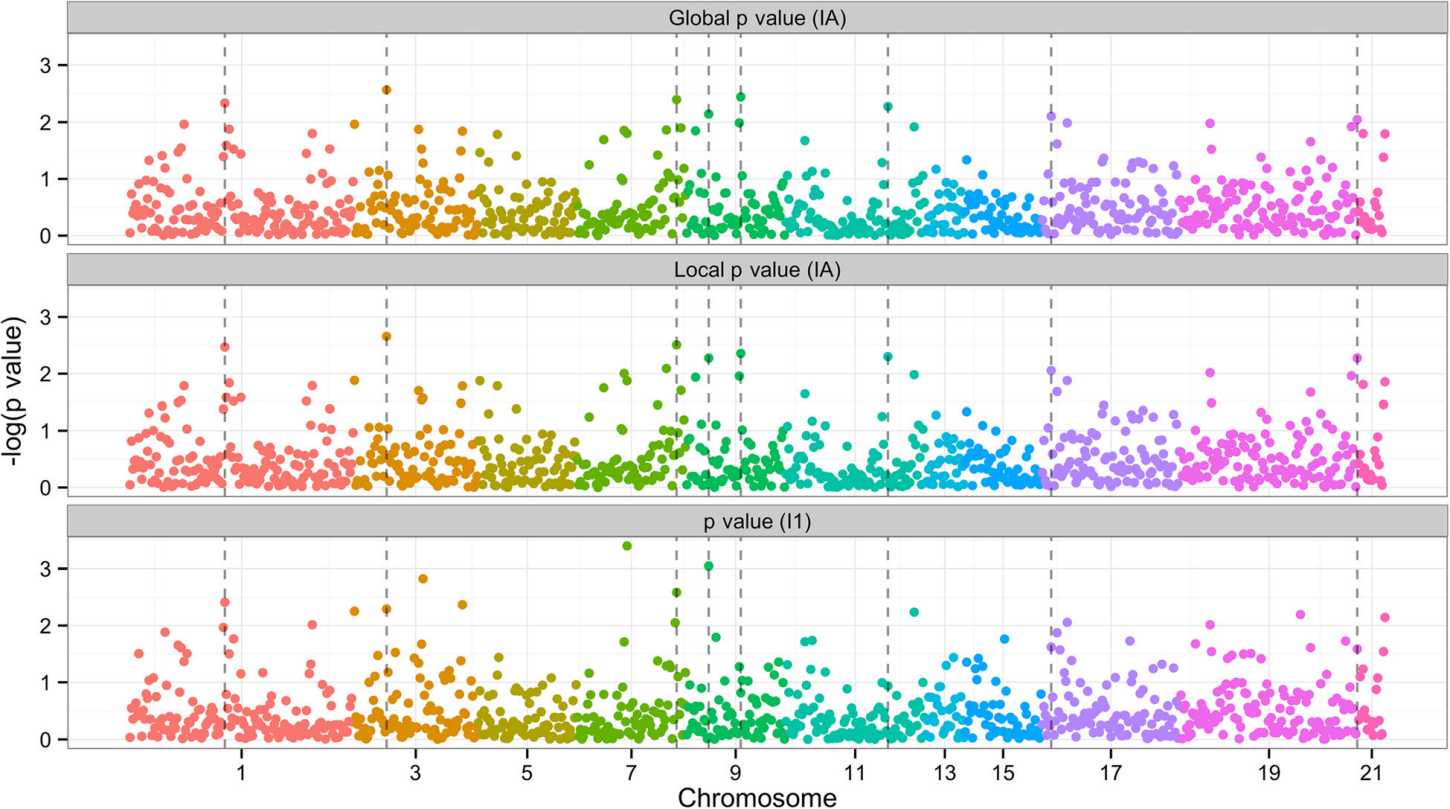
Manhattan plot of *p* values from LA-SPA and regular SPA. Important regions determined by the LA-SPA approach are marked by dotted lines. The global *p* value (IA) and local *p* value (IA) were calculated considering the ancestral origin of the region. The *p* value (I1) was calculated without considering the origin. Interesting regions are the last 4 dotted lines on chromosomes 9, 11, 17, and 19. These regions became relatively important only after the inclusion of local ancestry information

The number of rare variants and the 3 *p* values from the test statistic of each group of variants were retained after performing SPA twice (first for *I*_*A*_ and then *I*_1_; *I*_1_ was calculated for comparison purposes). *P* values are plotted in Fig. 1 and regions with relatively low *I*_*A*_ *p* values are listed in Table 1. We believe these regions contain additional, disease-related information and warrant more in-depth analysis despite that they are not significant when adjusting for multiple comparisons. This includes regions on chromosome 3 and 19, which, to our knowledge, have not yet been linked to hypertension or diabetes.

**Table 1:** Results of the LA-SPA procedure. Cytogenetic bands in each identified region already linked to hypertension or diabetes from the National Center for Biotechnology Information (NCBI) have been annotated in the last column

Number of rare variants in 500	Global *p* value (I_A_)	Local *p* value (I_A_)	*p* Value (I_1_)	Chrom	Position of first SNV in region	Position of last SNV in region	Cytogenetic bands in region already linked to hypertension
392	.0046	.0034	.0039	Chr1	70899547	76257984	1p31.1
434	.0027	.0022	.0051	Chr3	46964904	47452586	
402	.0040	.0031	.0026	Chr7	149519724	150490263	7q36.1
395	.0072	.0053	.0009	Chr9	37735625	71852004	9p11.1, 9q21.11
382	.0036	.0044	.1534	Chr9	117168826	120475298	9q33, 9q33.1, 9q33.2
409	.0053	.0050	.1154	Chr11	77961165	84997182	11q14, 11q14.1
395	.0079	.0088	.0239	Chr17	3631196	3962636	17p13.3
112	.0090	.0053	.0259	Chr19	59029075	59082725	

Regions with significant *I*_*A*_ test statistics but not significant *I*_1_ test statistics are also interesting since those regions became significant only after local ancestral information was taken into account. This includes regions on Chromosomes 9, 11, 17 and 19, which are the last four regions listed in Table 1. In the future, better results can be obtained by increasing the sample size so that the number of variants in each region can be reduced and the local ancestral information of each region can be calculated more accurately.

## Discussion

There are several points related to this approach worth mentioning. First, when performing PCA on all SNVs to measure global ancestry, we initially retained all of the loading scores of the leading principal components (PCs). To decide how many PCs to use, we assessed the stability of the loading scores by bootstrapping. We randomly sampled 10,000 SNVs with replacement 1000 times, and computed the scores based on each bootstrapped SNV set. We found that the loading scores of the first PC were highly correlated across different bootstrap runs. In contrast, the loading scores corresponding to other PCs were highly variable. We concluded that the first PC from genome-wide collections of SNVs consistently represents the global ancestry information.

Second, the decision to divide variants into regions of 500 was made after recognizing the tradeoff between genome resolution and the power to detect local ancestral information. Because local ancestry information is inferred from common variants, using regions of 500 SNVs yields an average of 100 common variants in each region that can be used to reliably infer local ancestral information. To judge the sensitivity of our method to this choice, we also performed the analysis with regions of 250 and 750 SNVs, which yield an average of 50 and 150 common variants respectively to infer local ancestral information. Of the 8 regions identified with regions of size 500, 3 regions on chromosomes 1, 7, and 17 were identified with regions of size 250 and 750. However, 3 regions containing cytogenetic locations associated with hypertension were identified only using regions of size 500 and not size 250 or 750. We believe that regions of size 500 are ideal as in regions of size 250 there is insufficient ancestry information and in regions of size 750 there is a loss in genomic resolution.

Finally, the choice of MAF = 0.05 as the cutoff for determining whether a variant was rare was somewhat arbitrary. We thought that 0.05 was low enough that the information contained within the rare variants would not be overwhelmed by the local ancestral information of the region. Considering multiple MAF cutoffs and observing whether the significance of the region changes would strengthen our method.

## Conclusions

The LA-SPA is a novel approach to variant selection that combines the power of admixture mapping with the power of SPA. When the subjects of a study are known to have come from an admixed population, exploiting ancestral structure can boost the signal of disease-related rare variants and allow for a more effective screening of the genome.
